# Predicting one-year left ventricular mass index regression following transcatheter aortic valve replacement in patients with severe aortic stenosis: A new era is coming

**DOI:** 10.3389/fcvm.2023.1130152

**Published:** 2023-04-04

**Authors:** Mohammad Mostafa Asheghan, Hoda Javadikasgari, Taraneh Attary, Amir Rouhollahi, Ross Straughan, James Noel Willi, Rabina Awal, Ashraf Sabe, Kim I. de la Cruz, Farhad R. Nezami

**Affiliations:** ^1^ Division of Thoracic and Cardiac Surgery, Brigham and Women’s Hospital, Harvard Medical School, Boston, MA, United States; ^2^ Bio-Intelligence Unit, Sharif Brain Center, Electrical Engineering Department, Sharif University of Technology, Tehran, Iran; ^3^ Mechanical Engineering Department, University of Louisiana at Lafayette, Louisiana, LA, United States

**Keywords:** statistical shape analysis, left ventricle remodeling, TAVR surgery, machine learning, Structural heart disease

## Abstract

Aortic stenosis (AS) is the most common valvular heart disease in the western world, particularly worrisome with an ever-aging population wherein postoperative outcome for aortic valve replacement is strongly related to the timing of surgery in the natural course of disease. Yet, guidelines for therapy planning overlook insightful, quantified measures from medical imaging to educate clinical decisions. Herein, we leverage statistical shape analysis (SSA) techniques combined with customized machine learning methods to extract latent information from segmented left ventricle (LV) shapes. This enabled us to predict left ventricular mass index (LVMI) regression a year after transcatheter aortic valve replacement (TAVR). LVMI regression is an expected phenomena in patients undergone aortic valve replacement reported to be tightly correlated with survival one and five year after the intervention. In brief, LV geometries were extracted from medical images of a cohort of AS patients using deep learning tools, and then analyzed to create a set of statistical shape models (SSMs). Then, the supervised shape features were extracted to feed a support vector regression (SVR) model to predict the LVMI regression. The average accuracy of the predictions was validated against clinical measurements calculating root mean square error and $R^{2}$ score which yielded the satisfactory values of 0.28 and 0.67, respectively, on test data. Our work reveals the promising capability of advanced mathematical and bioinformatics approaches such as SSA and machine learning to improve medical output prediction and treatment planning.

## 1. Introduction

With increasing prevalence, Aortic stenosis (AS) is the most common type of valvular heart disease (1). Severe AS represents a complex pathology which is not limited only to the aortic valve but also involves the left ventricular (LV) geometry and function. Left ventricular hypertrophy (LVH), defined by increased LV mass index (LVMI), is almost ubiquitous to severe AS and reflects the necessary myocardial compensation to chronic afterload elevation in attempt to maintain LV wall stress (2). This pathological remodeling process results in myocardial fibrosis leading to LV dysfunction and heart failure (3). Once even mild symptoms are present, failure to relieve elevated afterload leads to a mortality as high as 50% over 2 years (4). Unloading the heart with aortic valve replacement (AVR) tends to promote a decrease in LVMI and improvement in LV function, but the time course and degree of regression toward normality varies across patients (5,6). Long-term survival after AVR for severe AS is strongly related to the timing of the intervention in the natural disease course. Left ventricular ejection fraction (LVEF) and patients’ symptoms remain the main determinants of the timing of intervention in patients with severe AS in the guidelines (1). However, they are limited to global systolic function and fail to capture anatomical abnormalities, hindering their performance in risk stratification. It has been demonstrated that among patients with moderate or severe LVH treated with transcatheter AVR (TAVR), greater LVMI regression at 1 year is associated with lower mortality and hospitalization likelihood to 5 years (7). Despite its implications, these measures are largely absent from current symptom-based guidelines for the appropriateness of AVR (8,9). In recent years, several studies have attempted to leverage left ventricle statistical shape analysis to predict, classify, or analyze associated phenomena. For instance, left ventricle shape has been used to predict arrhythmic risk in fibrotic dilated cardiomyopathy (10), to predict response after cardiac resynchronization therapy (11), or to detect early signs of heart failure in congenital heart disease (12). However, left ventricle SSA has never been leveraged towards prediction of left ventricle mass index regression. In this research, we sought to evaluate whether three-dimensional (3D) left ventricular (LV) shape features, extracted from preoperative gated cardiac computed tomography (CT) scans leveraging statistical shape modeling (SSM), correlate with larger post-TAVR LVMI regression and identify a machine learning model to predict 1-year post-TAVR LVMI regression in patients with severe AS.

## 2. Materials and methods

**Figure 1:**
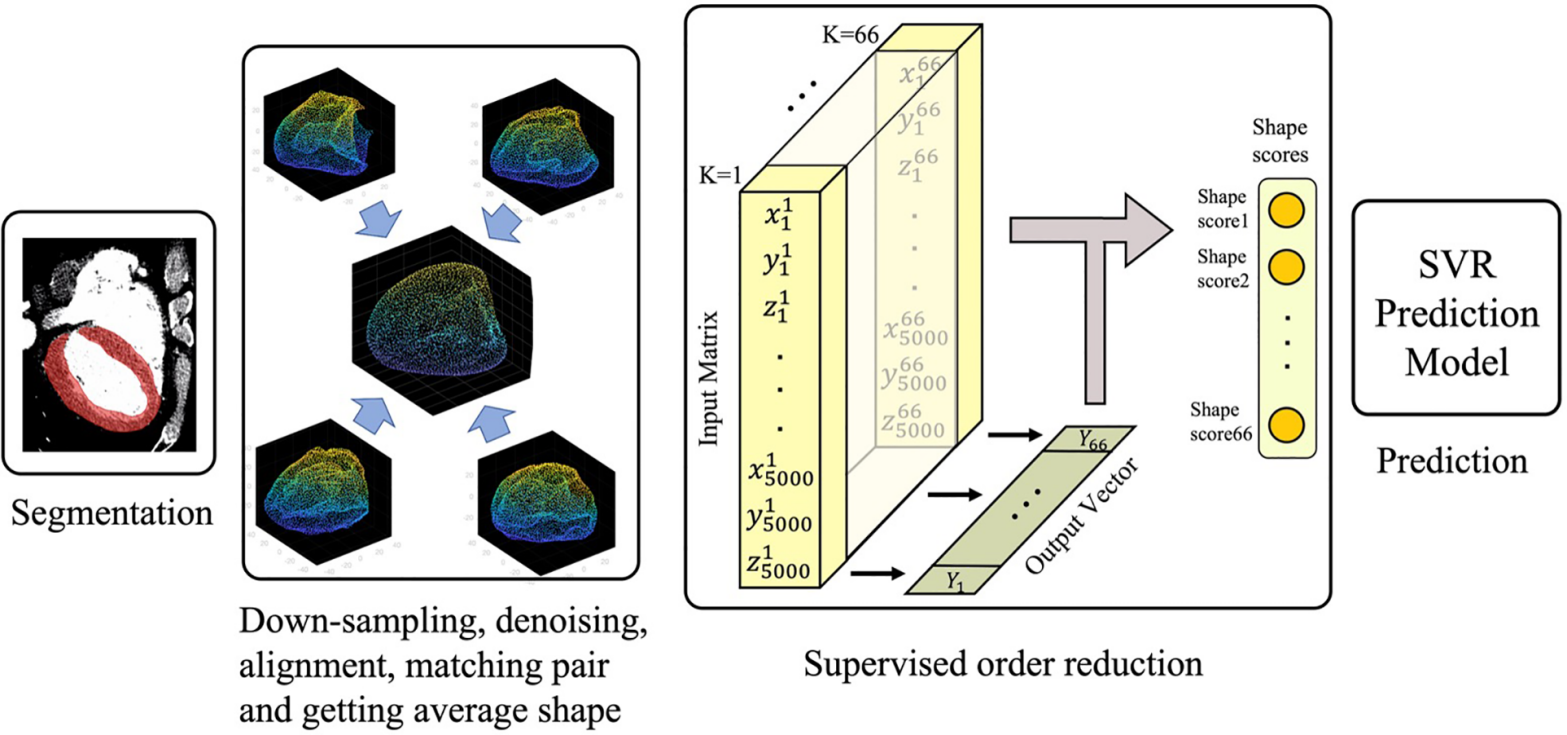
Schematic of the statistical shape analysis pipeline used for the outcome prediction.

The overall study design is illustrated in Figure 1. Briefly, the process was started with automatic segmentation of the left ventricles gated CT images as described in Section “Automatic segmentation.” The segmented 3D shapes then went through a pipeline of down-sampling, alignment, and order reduction (Sections “Shapes alignment” and “Left ventricle shape encoding”). The shape features extracted via order reduction (also called shape encoding) algorithm fed the Support Vector Regression (SVR) model which serves as our prediction machine. The predictor setup and the description of the input and output can be found in Section “Training the prediction model.”

### 2.1. Study population

We retrospectively identified all adult patients with severe AS who underwent TAVR at Brigham and Women’s Hospital (Boston, MA) from 2018 to 2020. Patients younger than 18 years with mixed aortic regurgitation or other concomitant valvular disease more than mild, history of ischemic heart disease, prior valve or coronary artery bypass graft surgery, and poor quality pre-TAVR gated cardiac CT images were excluded. The final study population included a cohort of 66 patients and the CT images were retrospectively collected in Digital Imaging and Communications in Medicine (DICOM) format with the approval from the local institutional review board. Table 1 demonstrates the baseline characteristics of these patients. The raw DICOM data were initially deidentified by an independent party converting images into a nearly raw raster data (NRRD) format. The LVMI regression percentage one year after the surgery is defined as:

(1) $$LVMIRegression=\frac{\;postLVMI-\;preLVMI}{\;preLVMI}$$

where preLVMI and postLVMI refer to the LVMI before and after the TAVR, respectively. The pre and postLVMI were calculated from transthoracic echocardiography as described before (13)

**Table 1:** Patients’ Characteristics for the studied population.

Characteristics	No. (%) or mean ± SD
Female	32 (48)
Age	76.73±9.14
NYHA functional class	
I/II	48 (73)
III/IV	18 (27)
Left ventricular ejection fraction (%)	61.83±8.11
Diabetes	19 (29)
Hypertension	59 (89)
Severe COPD	3 (4.5)
STS Risk Score	
Low	23 (35)
Intermediate	39 (59)
High	4 (6)
PreLVMI (kg.m${}^{2}$)	90.40±25.18
One-year LVMI	89.51±22.96

### 2.2. Automatic segmentation

**Figure 2:**
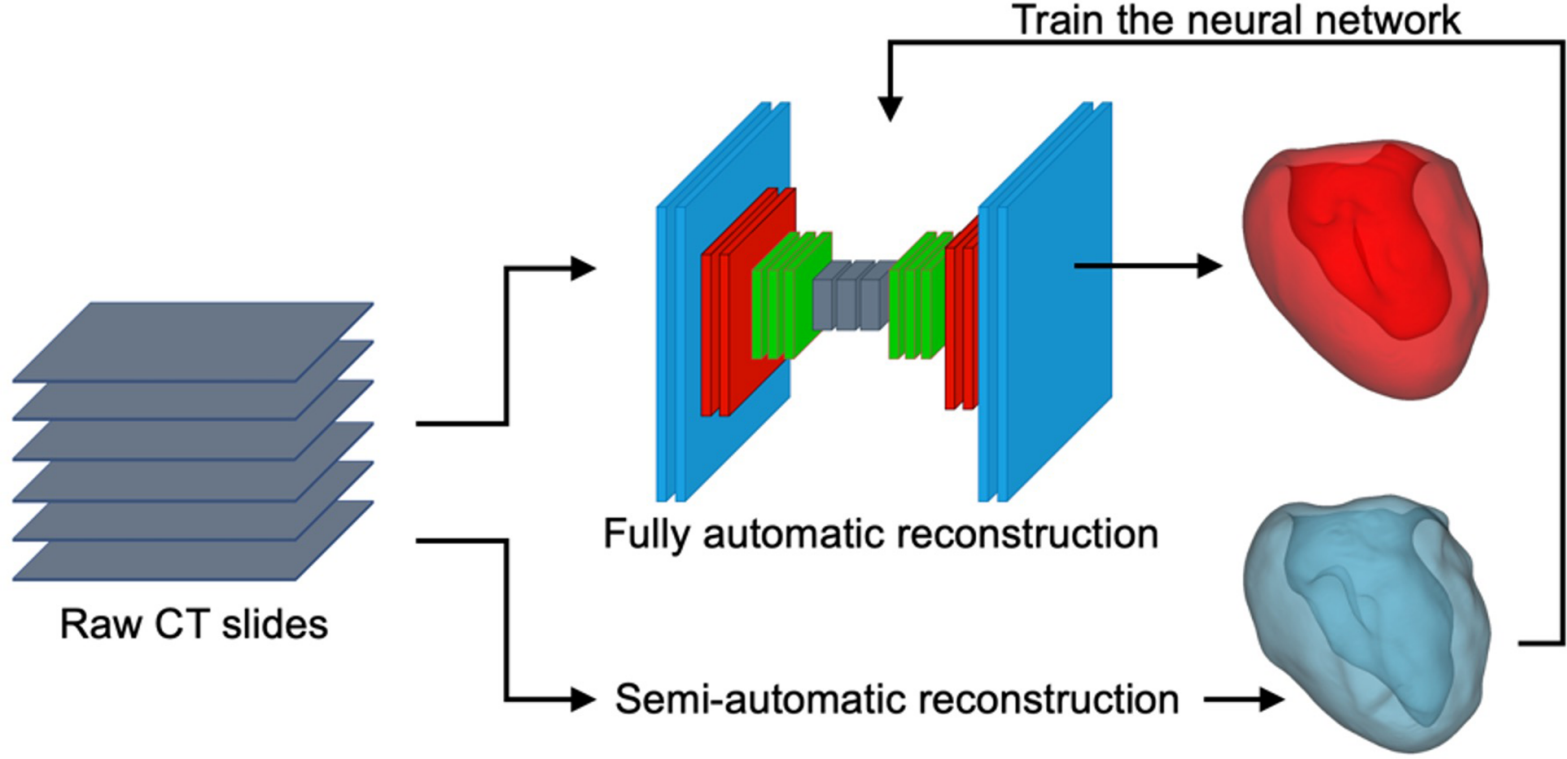
Schematic representation of the U-Net neural network, semi-automatic segmentation of the LV using 3D-Slicer (blue), and a sample AI-predicted reconstructed LV geometry (red).

To reconstruct the digital twins of studied LV, a convolutional neural network (CNN) algorithm using a U-Net structure was trained and validated using LV masks generated by semi-automatic segmentation of 35 cases with the open-source 3D-Slicer package (Figure 2). The semi-automatic annotations for training and testing purposes were done by two independent clinical experts who annotated randomly selected patients. The annotations were later revised by an independent operator, blind to the assigned clinical annotators. Adopting such an approach, we strived to minimize the operator dependency and bias in our segmentation approach. Future steps were handled by the trained and tested neural network which was automated and included no operator interference.

**Figure 3:**
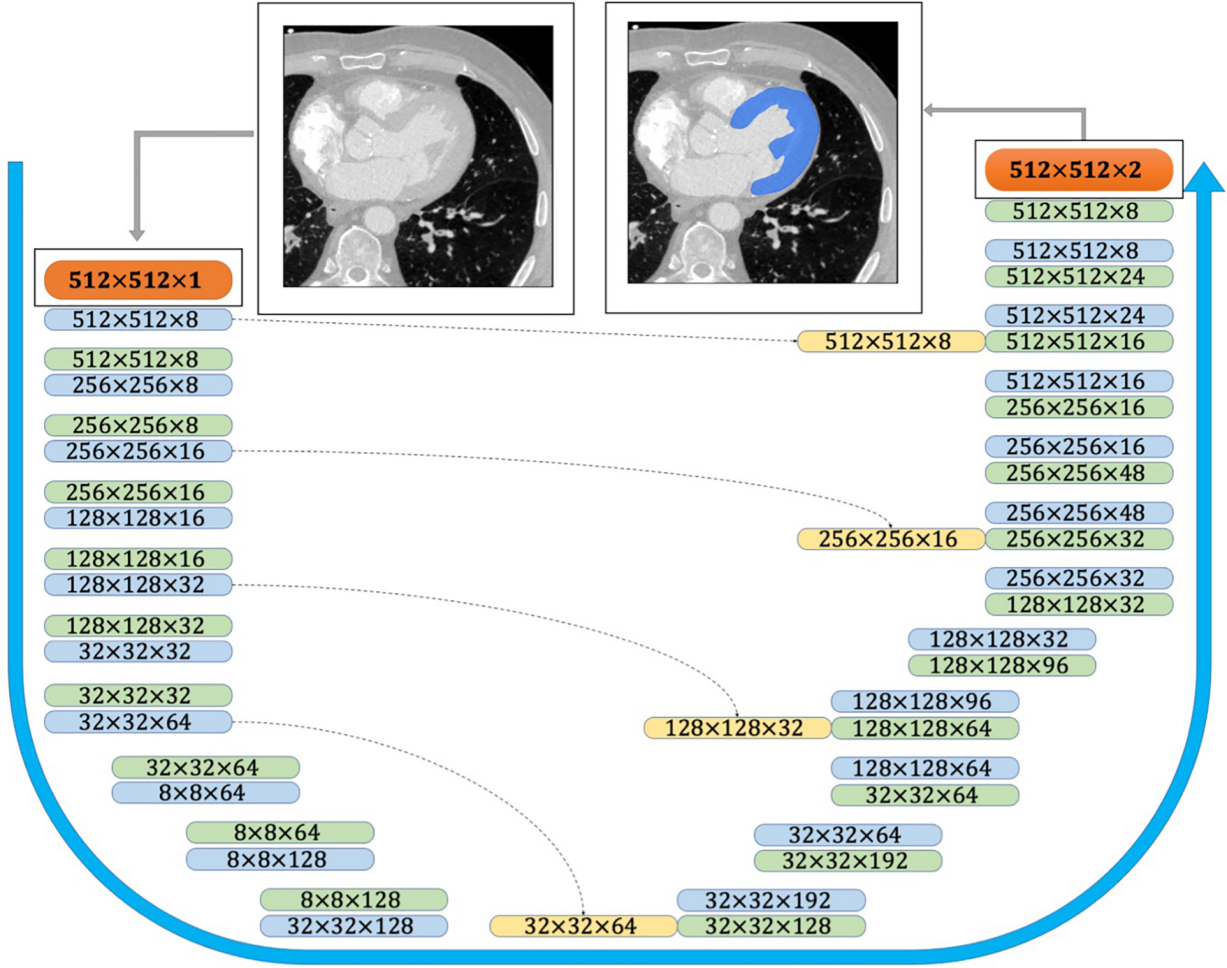
CNN architecture used for semantic image segmentation. The input and output images are shown as orange boxes. The inputs and outputs of the CNN layers are shown as green and blue boxes, respectively. The yellow boxes represent the copied tensors, and the blue arrow indicates the flow of the CNN model from raw input image to the binary segmented output image.

The provided CT scans and the corresponding labels were initially normalized and then cropped/padded to 512–512 pixels. The available training data was augmented using scaling and rotation to generate a sufficient training set. The Keras python library was used to perform the deep learning powered automatic segmentation due to its competent performance and structure. The U-Net architecture comprises two major components (Figure 3): (i) encoder which includes the convolutional and pooling layers to extract the important features of the input images; (ii) decoder which consists of convolutional layers and up-sampling to build the predicted output segmentation (14). In addition to the network structure, multiple network hyperparameters were tuned to obtain high accuracy and shorter training time. The CNN model was trained on a Quadro RTX 6000 GPU including 4,608 Cuda cores and 24 GB GPU memory.

To gain robust performance, the model was trained using five subsets to provide five variations of the deep learning model. During the training, fusion of the five candidate labels was obtained using majority voting (ensemble training) (14). The predicted segmentations obtained from the deep learning model were stored as NRRD files, and additional image processing steps were performed. First, a closing operation, the erosion of the dilation of the label, was performed with a cubic structuring element of 5 voxels in diameter to remove small holes within the volume. To smoothen the generated label, a median filter utilizing a ball shaped footprint of 5 voxels was applied. After the image has been smoothened and small holes removed, the largest connected component was isolated, removing any unwanted islands. All models were trained and evaluated using five-fold cross-validation on the training set. The average value of dice loss function was 0.96 indicating promising efficiency, visually validated by comparing the predicted models with the geometries reconstructed by skilled clinicians. A marching cube and a subsequent Laplacian smoothing operation is performed on the labels to create the smooth 3D geometry which is then converted to an STL file. We experienced that, with sufficient resolution, the impact of marching cubes operation on the intracavity volume was minimal. Such characteristics justify the frequent use of this technique for meshing in platforms such as with the VTK python API that we also utilized herein. Using the Laplacian smoothing operation, we controlled, and experimentally optimized, two main parameters in our process namely the number of iterations and the relaxation factor. The smoothing operation adjusted the coordinates of every individual vertex based on the average of the connected vertices, while the amount of displacement was controlled by the relaxation factor.

### 2.3. Shapes alignment

The produced STL files typically comprise a quarter of million points. Processing such huge point clouds is computationally expensive and practically unnecessary. Moreover, registration algorithms are more likely to inaccurately align these shapes due to local minimization issues in the internal iterative optimization process (15). Another advantage of down-sampling process is denoising and smoothing the unrealistic spikes of the generated shapes. We used Open3d package in Python to down-sample and convert the point clouds into polygon (ply) file format, where each individual shape consistently contained 5,000 points in the 3d space building a 15,000-length location vector for each shape. The down-sampling process is theoretically reported to be unbiased with guaranteed uniformity. Preliminary tests of varying down-sampling efforts returned minimal change in the SSA outcome and mass center locations.

Before starting the alignment process, all the point clouds were shifted such that the mass center of each shape was transferred to the origin (x=y=z=0). This accelerated the iterative optimization process in the alignment algorithm and can be compared to the traditional data normalization in pre-processing input data in machine learning routines. In the next step, the iterative closest point (ICP) algorithm was applied to align the shapes (16,17). ICP aligns the shape p with respect to the reference shape q by applying the scale s, rotation matrix R, and the translation (shift) transformation T on it. The method aims to minimize the global error E.

(2) $$E=\sum_{i=1}^{n}\|e_{i}{\|}^{2}$$

between the shapes p and q, wherein the residual error $e_{i}$ is defined as

(3) $$e_{i}=s.Rp_{i}+T-q_{i}$$

in which $p_{i}$ and $q_{i}$ are corresponded points in shapes p and q. In this study, we set s=1 to conserve the size of left ventricle as a clinically important feature. Traditionally in SSA, the reference shape is randomly selected. Here, we tried to enhance the approach by selecting the shape with surface and volume closest to the averages of the whole shapes as the reference shape. Specifically, the reference shape was selected to be the one which minimizes

(4) $$\frac{s-\bar{s}}{2}+\frac{v-\bar{v}}{2}$$

where s is the surface area for each shape, v is the volume, and overlines indicate the mean value. After aligning all the shapes with the reference shape, the location vectors need to be reordered. A point cloud noted as $X^{\left(k\right)}$ can be represented by its 5000×3 location matrix defined as

(5) $$X^{\left(k\right)}=[x_{1}^{\left(k\right)},\ldots ,x_{n}^{\left(k\right)},\ldots ,x_{N}^{\left(k\right)}{]}^{T}\in R^{5000\times 3}$$

assembled from the coordinates of point k defined by a 3×1 size vector $x_{n}^{\left(k\right)}$ with a total number of N (5000) points. The rows of the location matrix were reordered such that the ith row (i=1,…,5000) in the location matrix of each individual shape was matched with the point addressed in the same (i.e., ith) row of the location matrix for the average point cloud. The average shape was then simply calculated as

(6) $$\bar{X}=[{\bar{x}}_{1},\ldots ,{\bar{x}}_{n},\ldots ,{\bar{x}}_{N}],\;\text{where}\;{\bar{x}}_{n}=\frac{1}{K}\sum_{k=1}^{K}x_{n}^{\left(k\right)}$$

in which ${\bar{x}}_{n}$ is a point on the mean shape and K is the number of the shapes. Once the mean shape was computed, the shapes were realigned to this new mean shape to arrange them along the newly defined reference. The last step before order reduction (also called encoding) is calculating the deviation vector of ith shape with respect to the average shape by subtracting the ith location vector from the average shape location vector. By that we aimed to remove the common information among the individual clouds and facilitate the machine steps.

### 2.4. Left ventricle shape encoding

Shape encoding (See Figure 1 for a schematic view) aims to represent a shape by a small number of scalar values far less than the order of the location matrix, which contains 15,000 entries in our case. High dimensional regression problems suffer multicollinearity, and the encoding methods are employed to alleviate it. There are several methods available for shape encoding, including principal component analysis (PCA), partial least squares (PLS), autoencoding, and sparse coding (18). Notably, PLS is the only *supervised* order reduction method among all mentioned approaches including the popular method PCA. In particular, while the PLS components are chosen so as to describe most possible of the covariance between the input and output datasets, PCA and other unsupervised order reduction methods only concentrate on the variance of the input. More specifically, PLS aims to incorporate information on both input and output in the definition of the extracted components. While PCA is highly popular and extensively used, we tried both PLS and PCA in the current study to compare their accuracy in the outcome prediction to one another.

At the first step of order reduction, the 5000×3 location matrix was reshaped into a 15,000×1 location vector. In this structure, the shapes are defined as

(7) $$X^{\left(k\right)}=[x_{1}^{(k)T},\ldots ,x_{n}^{(k)T},\ldots ,x_{N}^{(k)T}{]}^{T}\in R^{15,000\times 1}$$

Shape $X^{\left(k\right)}$ can be approximated by the summation of the mean shape and a linear combination of the first m shape modes, given by

(8) $$X^{\left(k\right)}≅\bar{X}+\sum_{k=1}^{m}a_{km}U_{m}$$

where $a_{k}m$ is the mth shape score of $X^{\left(k\right)}$ and $U_{m}$ is mth shape mode. Herein, the input matrix X is of size 66×15,000 in which the ith row contains the location vector of the ith patient’s LV point cloud, and output Y is a 66-length vector containing the LVMI regression of the patients.

### 2.5. Training the prediction model

While classifiers can simply divide the patients into groups with relatively higher or lower level of LVMI regression, prediction of the LVMI regression will be of more clinical value. Thus, we here focus on leveraging regression models to map extracted shape features directly to the LVMI regression. Since the PLS built-in predictor machine uses the simple linear regression algorithm, we extracted the principal components of PLS and employed a more powerful prediction method called support vector regression (SVR) (19). Such a superior predictor provides advanced tools such as cross-over validation and nonlinear regression functions to enhance the prediction accuracy. SVR is a regressor variant of support vector machine (SVM) which describes nonlinear relationships between input and output. We measured the regression accuracy by calculating the root-mean-square error (RMSE) and $R^{2}$ score. *RMSE* is calculated as

(9) $$RMSE=\sqrt{\frac{\sum_{i=1}^{n}\|y(i)-\hat{y}(i){\|}^{2}}{n}}$$

where n is the number of data points, y(i) is the ith measurement, and $\hat{y}(i)$ is its corresponding prediction. Since *RMSE* is not scale invariant, it is commonly used over normalized data as we did through this paper. $R^{2}$ score is a statistical measure that is used to assess how much variation of an outcome is explained by the independent variables in a predictor model. It can be defined as

(10) $$R^{2}=1-\frac{\text{Unexplained\textbackslash{}; Variation}}{\text{Total \textbackslash{};Variation}}$$

or, in a more mathematical presentation, as

(11) $$R^{2}=1-\frac{\sum_{i=1}^{n}(y_{i}-{\hat{y}}_{i}{)}^{2}}{\sum_{i=1}^{n}(y_{i}-{\bar{y}}_{i}{)}^{2}}$$

in which n is the number of predicted values, $y_{i}$ is the ith output real value, ${\hat{y}}_{i}$ is the estimated value of $y_{i}$, and ${\bar{y}}_{i}$ is the average value over the outcomes. Values of $R^{2}$ score closer to 1 reflects higher accuracy in predicting outcomes, while lower score indicates that the accuracy of the prediction model is simply estimating all outputs by merely using the total average, ignoring any input data. A negative $R^{2}$ score however would indicate that the model’s prediction accuracy is even worse than an estimating by average.

## 3. Results

### 3.1. Statistical shape model (SSM)

**Figure 4:**
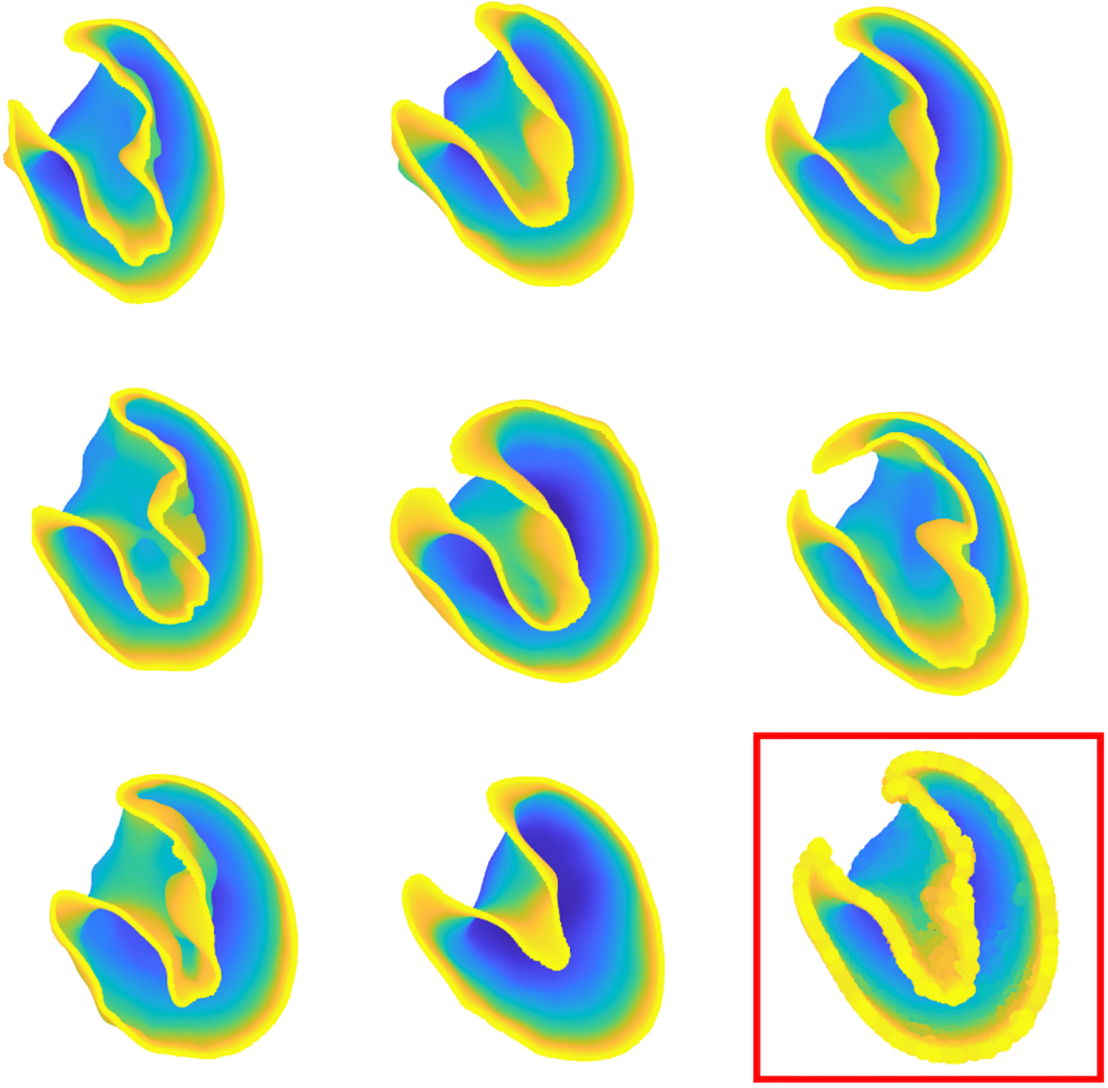
Some of 66 patient-specific left ventricle shapes after processing and preliminary operations. The template shape is indicated in the bottom right of the figure.

We successfully developed CT-image based SSMs to perform the prediction task based on extracted shape features. LV shapes were generated after denoising, down-sampling, and alignment along the average shape (Figure 4). Moreover, SSMs allowed us to analyze LV shapes by visualizing the most important modes identified by the PLS, as followingly described.

### 3.2. Shape encoding and features extraction

**Figure 5:**
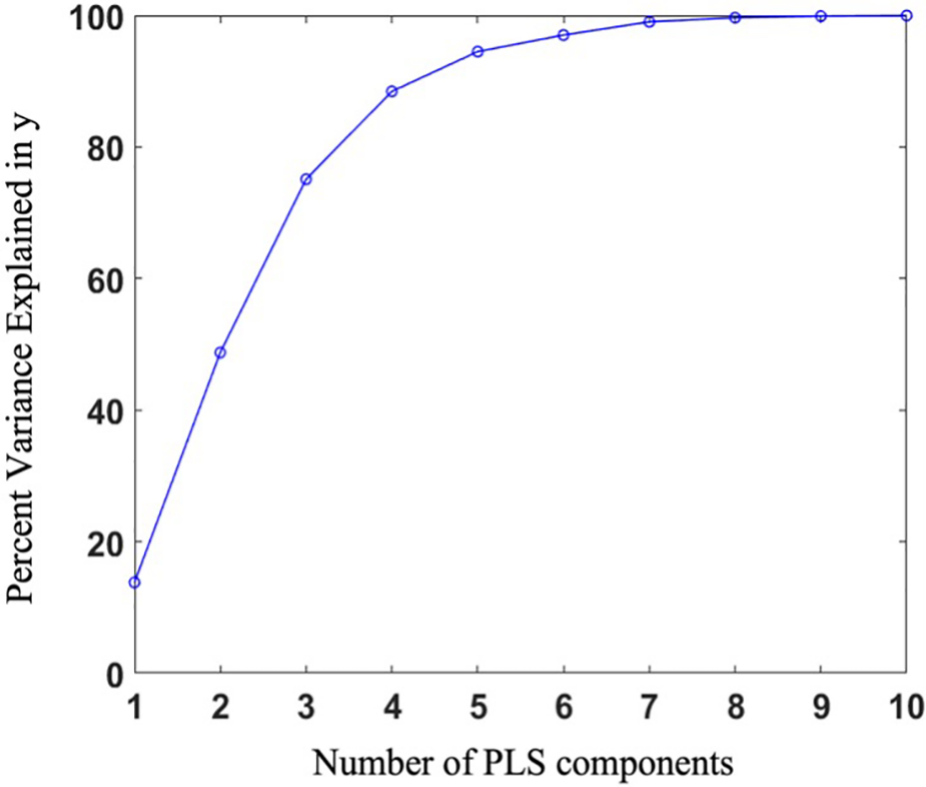
Percentage of variance explained in the outcome as a function of the number of shape components.

PLS regression was employed to extract the shape scores for each case along with the global shape modes. The variance explained in the response was captured by each of the first 10 PLS modes, and their cumulative accounted variance was quantified (Figure 5). Though the first three modes together capture more than half of the output variation, we decided to predict the outcome using the first five modes which contain nearly 95% of the output variance explained by the shape components.

**Figure 6:**
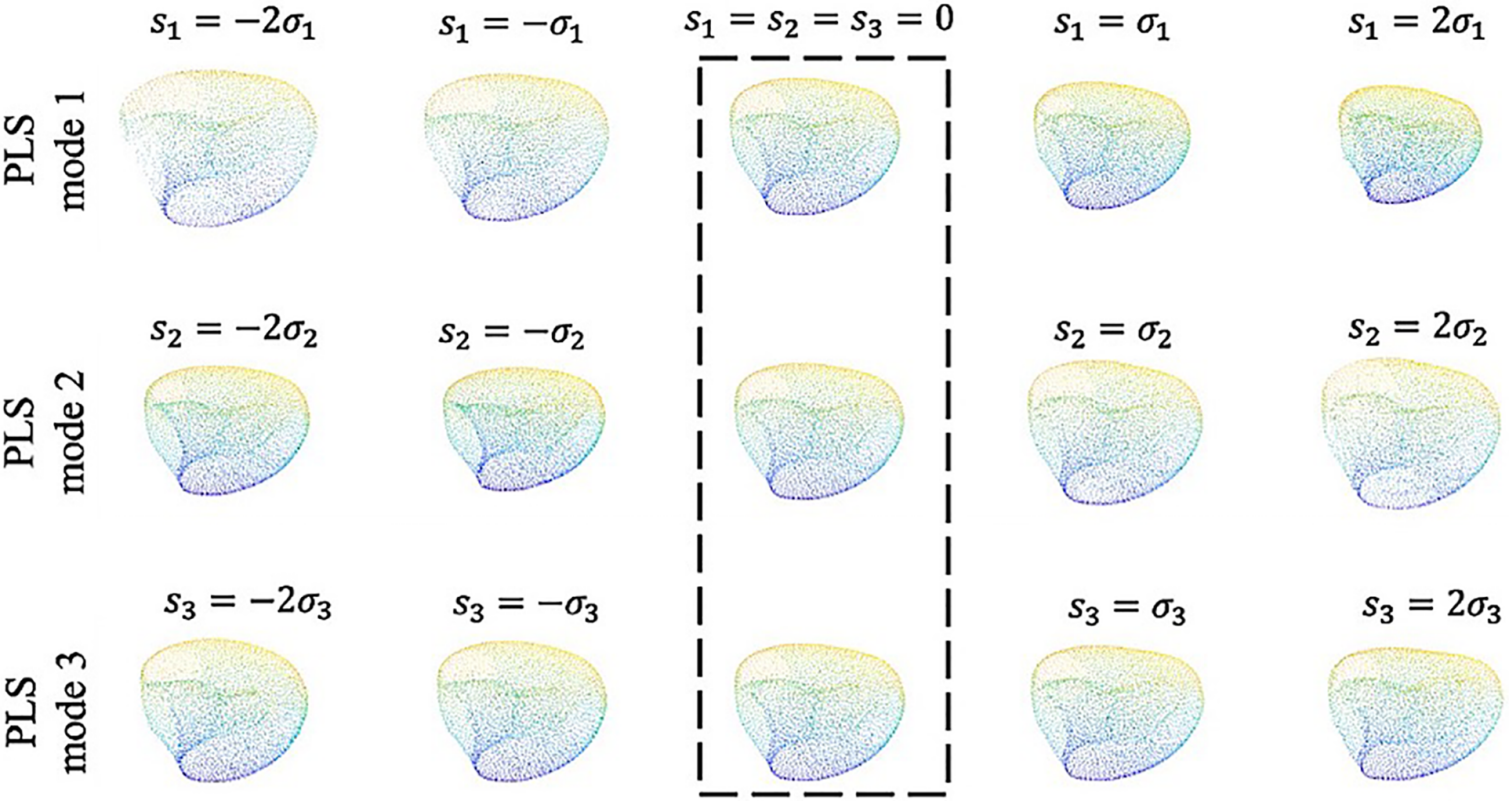
The first three shape modes which together convey 74% of the total covariance between the input and output. Along each row from left to right the shapes are ordered by increasing shape score as $-2\sigma_{i}$, $-\sigma_{i}$, 0, $\sigma_{i}$ and $2\sigma_{i}$, respectively. The template shape (s=0 along each mode) is indicated by the dashed box.

Visualizing the effects of variation in the first three principal scores allows to coarsely connect the mathematical measures of SSA analysis to the anatomical presentation and morphological features (Figure 6). Building such an analogy reveals how shape analysis comprehensively embodies the anatomical characteristics of clinical cases as a whole and outfitted for outcome prediction without being explicitly limited to first-hand, 2D measurements (such as diameter) that might not necessarily bear any predicting power. Accordingly, comparing the visual presentation of principal shape variation speculates that the first component mainly describes the volumetric size of the LV, the second seemingly explains the variations of the chamber’s volume, and the third addresses spherical to ovoidal conversion (otherwise called aspect ratio metric in some studies).

### 3.3. Prediction

**Figure 7:**
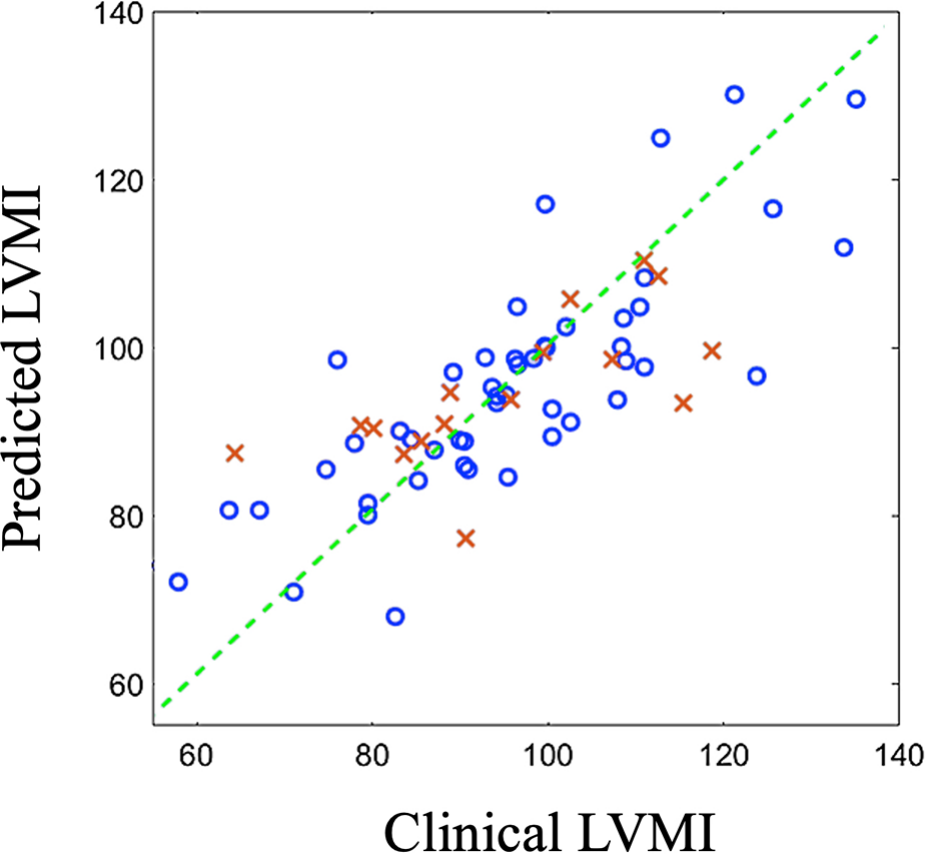
Predicted outcomes (vertical axis) vs clinically reported outcomes (horizontal axis) for train data (blue circles) and test data (red crosses). LVMI: Left ventricle mass index.

The predictor model is an SVR regressor with linear kernel function. We randomly selected 15% of cases as the test data and used the remaining samples (85%) to train the predictor model. The random splitting of the original data is essential for providing an unbiased train and test data set especially for this small cohort. Our initial analysis of bootstrapping the training and test data also demonstrated minimal changes in prediction performance. The developed model successfully performs to predict the output with great accuracy (RMSE=0.2819, $R^{2}\;\text{score}=0.68$), confirming the great load of latent information existed in the medical images and generated shapes (Figure 7). We report that predictions based on PLS components exhibit higher accuracy than PCA-based predictions (RMSE=0.37, $R^{2}\;\text{score}=0.57$) as expected.

**Figure 8:**
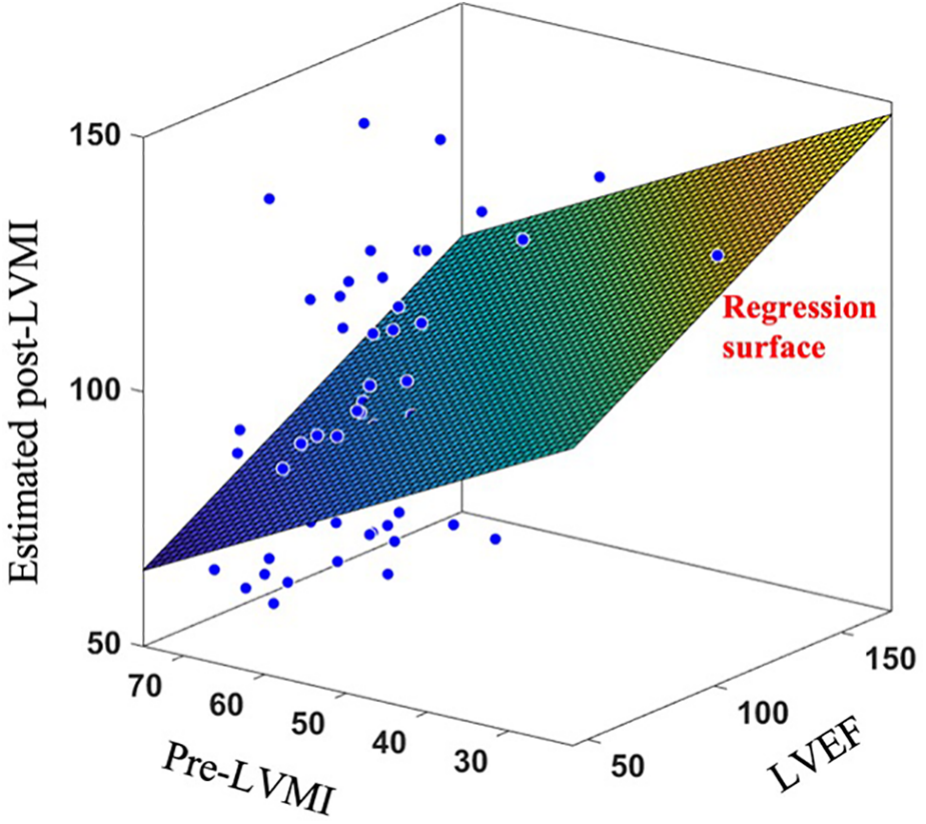
Applying linear regression surface to estimate post-LVMI based on clinically measured LVEF and pre-LVMI.

To further exhibit the superiority of the proposed shape-based method over currently available clinical decision making algorithms, we have also fit a multivariate regression model to estimate post-TAVR LVMI regression percentage at one year based on two key-factor values which are routinely reported in clinics; namely the pre-operation LVMI (preLVMI) and left ventricle ejection fraction (LVEF). While the prediction power of linear regression provided here is not expected to be even close to the performance of an AI-based advanced method like SSA, the comparison here aims to quantitively highlight the superiority of modern methods over the traditional approaches which are currently common in clinics. The regressor equation assumes the following form

(12) $$y(t_{1},t_{2})=p_{0}+p_{1}t_{1}+p_{2}t_{2}$$

in which y is the estimated LVMI regression, $t_{1}$ and $t_{2}$ are preLVMI and LVEF respectively, and $p_{0}$ , $p_{1}$ and $p_{2}$ are the regressor parameters to be found such that the summation of the squares of the estimation error be minimized. The regression result is shown in Figure 8, and the parameters are found as

(13) $$p_{0}=113,\;p_{1}=0.32,\;p_{2}=-0.82$$

While the role of $p_{0}$ is to simply move the outcomes’ center of mass from the origin to the mean of LVMI regression, the absolute values of $p_{1}$ and $p_{2}$ reflect the impact and power of their associated clinical values in the outcome prediction. In this case, we observed that the role of LVEF in estimation of LVMI regression is more than double that of preLVMI emphasizing the importance of LV function in predicting post-TAVR cardiac reverse remodeling. The prediction assessment values (RMSE=0.42, $R^{2}\;\text{score}=0.1$) however reveals that the proposed linear regression is inferior in the performance compared to the results achieved by the proposed SSA. We additionally investigated whether adding this pair of data to the shape scores vector may improve the prediction accuracy; however, no significant improvements were observed.

Another hypothesis that we explored was whether different time-points of the cardiac cycle become a superior instance to collect data in terms of predicting utility for the LVMI. Performing three independent sets of SSAs with LV shapes generated from image sets at systolic, end-systolic, and end-diastolic of the cardiac cycle (defined as 25%, 35% and 75% of the cardiac cycle, respectively), we observed negligible differences in the prediction performance (RMSE values of 0.28, 0.26 and 0.29 for systolic, End-systolic and End-diastolic sets, respectively).

## 4. Discussion

Machine learning tools and advanced mathematical models are increasingly being leveraged to improve diagnosis, inform therapy planning, and enhance clinical care. A plethora of image-based tools and reduced order models have recently been offered to extract insightful measures from clinical images. Herein, we offer a fully automatic ML-SSA approach to accurately predict the LVMI regression following TAVR in patients with severe AS leveraging the baseline geometry of the left ventricle.

The current evaluation of patients with aortic stenosis accounts for changes in valve size and function and recommends intervention when the patient becomes symptomatic, or the LV function deteriorates (1). However, multiple studies have demonstrated that LV remodeling from longstanding elevated afterload as well as ischemia-induced myocardial fibrosis and LV diastolic dysfunction are risk factors for inferior survival after aortic valve replacement (9,20). Yet, their contribution to the risk stratification algorithms and timing of intervention is a matter of controversy. In the current practice, LV remodeling is defined by preLVMI which is an estimate from 2D transthoracic echocardiography and perhaps would not efficiently represents the effects of chronicity and severity on the left ventricle.

To properly characterize the LV shape and predict the cardiac remodeling, we developed a machine learning model with a novel structure that consists of several modules including digital twin generation (automatic segmentation using deep learning and geometry reconstruction), shape analysis (down-sampling and denoising, shape alignment, matching points pairs, and order reduction), and the prediction model. Our promising results reveals that an abundance of hidden information is buried within the clinical images and geometrical shapes of LVs which are not readily available nor are captured in the current clinical and imaging assessments.

SSA is a mathematical technique that enables identification of shape features that correlate strongly with an outcome of interest. Particularly, cardiovascular disorders have been an insightful focus of SSA by revealing close correlation between cardiovascular function and geometry and variations thereof. SSA methods are generally employed to provide clinical insight in two major ways. The most popular application of SSA is classification, in which shape features are leveraged to organize the subjects into two or more groups. For instance, the differences in the first bunch of PCA components have been used to distinguish aneurysmatic and non-aneurysmatic LV shapes (21). We have also previously studied hemodynamics in patients with ascending aortic aneurysm using SSA routines (22) and disclosed that shape numbers, in contrast to first-hand geometrical metrics such as maximum aortic diameter, are better predictors for the thoracic ascending aortic dissection risk. Moreover, SSA could also serve to estimate a continuous value outcome rather than a binary classification, so called a regression problem. As a case in point, a model has recently been developed to estimate the stress distribution in aortic arch via the aortic segmented 3d shapes (14). While the input set of the predictor model in this type of work is the aorta shapes’ extracted features, the output is also a set of algorithms to reconstruct the outcome of interest, there the 2d-image of stress heat map. Thus, order reduction in regression models is not only being used to summarize the predictor model input (e.g., aorta shape in (14)), it is also leveraged to estimate the quantified measure of interest (e.g., heatmap of the stress distribution over the aorta in (14)). These methods have been leveraged to address different problems based on CT, echocardiography and MRI images (23,24). Notably, the models originated from CT were found to exhibit a steady quality with respect to spatial resolution while being free of user bias in terms of image quality.

A literature survey on LV segmentation reveals that most of published image processing attempts and SSMs generate and later employ a rather smooth ventricular inner wall neglecting the details of papillary muscles and trabeculae (25). Papillary muscles morphologically create some swelling zones at the site of their connection to the LV wall, while the trabeculae, conversely, manifests as bundles or pieces of muscle that extend into the ventricular chamber. We originally hypothesized that inner site of LV may play a key role in the estimation of LVMI regression which would render our approach more sensitive to and inclusive of the details of the geometry of the left ventricle and its inner wall. We posited that even those minor changes to the morphology of inner wall may embed some latent information worthy of prediction power and contributing to the shape analysis. Thus, we decided to train and develop our segmentation tool with utmost fidelity to the inner wall geometry and minimized the aggressive smoothening attempts that were otherwise practiced.

**Figure 9:**
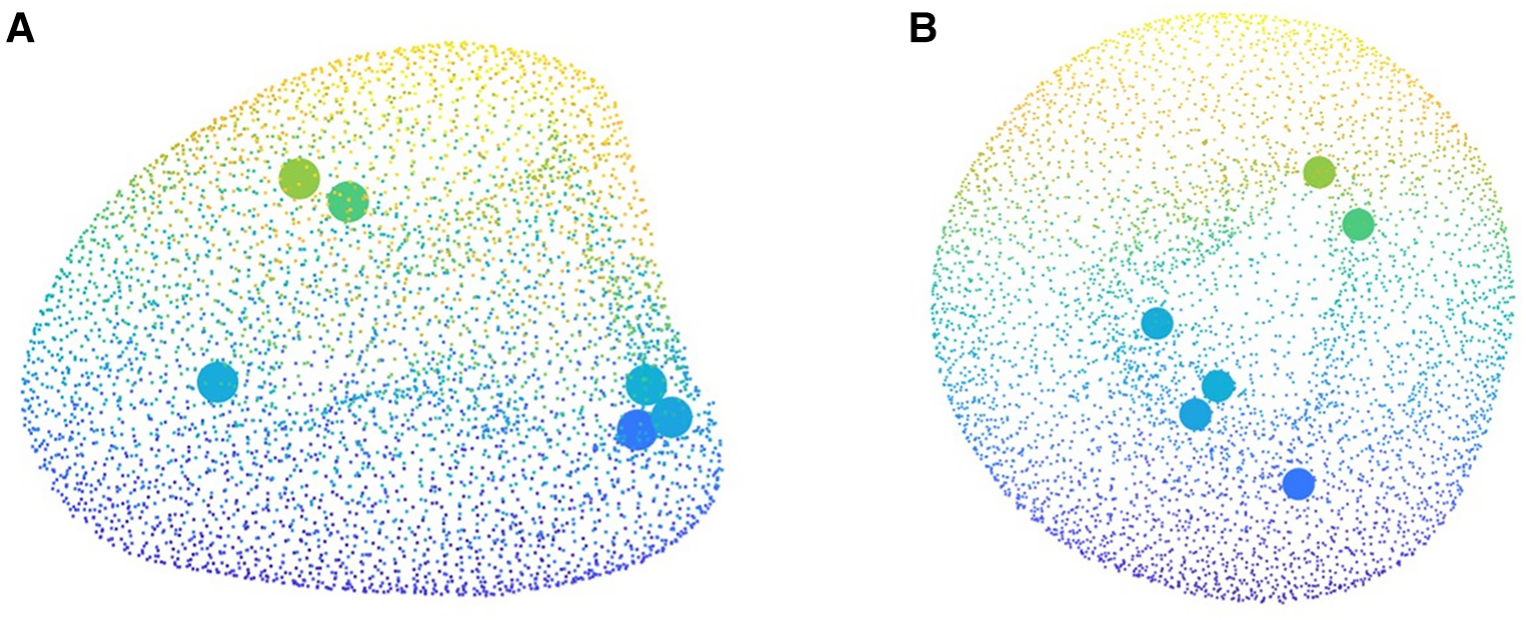
Critical points of heart contributing to the cardiac remodeling captured by the variable importance in projection (VIP) score which are all located on the internal wall of the left ventricle.

Another important utility of PLS method is providing variable importance in projection (VIP) scores. VIP scores estimate the importance of each variable in the projection used in a PLS model and is often used for variable selection. Traditionally, a variable with a VIP score close to or greater than 1 can be deemed important in a given model, while the variables with VIP scores significantly less than 1 are strong candidates for exclusion from the model. VIP scores can be interpreted simply as “the higher, the more important.” It might be clinically insightful if the mathematical approach also highlights the spatial location within the anatomy with more contribution to the outcome. Such an input could be properly extracted by identifying the points in the average LV point cloud possessing the highest VIP scores (Figure 9). Remarkably, these points for our study, with VIP values above 2.6, all lie on the inner wall of the left ventricle chamber. We observed that majority of these points, i.e. half of them, were located near the aortic valves while two were positioned near mid-anterolateral zone and the remaining critical point was located near the LV apex. This observation, from one side, is in agreement with the fact that the internal wall of the LV is more involved in remodeling rather than the left ventricle external surface which is limited by the pericardium. In addition, this preliminary remark suggests further focusing on selected spatial zones in the anatomy that manifest major contribution to LVMI opening doors for future research. Such an observation as well justifies our decision to include even small details of the LV inner surface and avoid the traditional, aggressive smoothening of the geometry that might otherwise have affected the shape analysis.

Our results demonstrated that adding preLVMI and LVEF would not improve the accuracy of the SVM model. Although the independent association of LV function and remodeling with post-AVR survival has been evaluated in the previous studies, our findings may suggest that the information related to LVMI regression provided by this pair is either poor and/or already conveyed in the geometry of the LV. In other words, these observations reveal that adding those components do not provide any additional information more than those provided by LV shape to enhance the prediction accuracy. Furthermore, the independency of the results from the time points of cardiac cycle can be readily interpreted by considering the fact that order reduction and prediction are based on the deviation vectors of the shape calculated by subtracting the coordination of each point in a particular shape from its matched pair in the mean shape. In other words, the reformation of left ventricle at each time point of cardiac cycle than another time point can be seen as a change in the mean shape and therefore is eliminated in producing the deviation vectors.

Discussing different clinical components of LV remodeling analysis, there exists a number of anatomical features such as wall thickness that are measured via follow-up imaging and recruited to assess remodeling occurrence and severity. Another benefit for using SSMs is the ease of quantifying those clinically utilized components at baseline from digitized cases allowing the study team to assess their evolution as well as predicting power for outcome. Though considering the shape in entirety would inherently include the information for geometrical features such as wall thickness, it is still feasible to add any anatomical feature of choice exclusively to the analysis pool. Once the geometrical feature is properly quantified, PLS would capture those properties of the anatomy and implement them for clinical prediction.

When a prediction task is performed by components extracted via order reduction methods such as PCA or PLS, one hyper-parameter that needs to be set is the number of first components to which the model is fed. Picking a small set of the first components can result in missing a non-ignorable piece of information in the original data. On the other hand, selecting a large number of components will result in an unnecessary complexity (note that most of the information are captured in the first components and in most cases, including ours, the first 10% of the components convey nearly 98% of the shape data) and, more importantly it may cause overfitting problems when the size of available dataset is not large enough. To find the best value of this hyper-parameter, we performed the prediction in different scenarios of $n_{c}=3,4,5$ and 6 when $n_{c}$ denotes the number of picked components. As a rule of thumb, a reliable method to check if a predictor machine is working well in the sense of overfitting is comparing the $R^{2}$scores for training data and test data. In a properly-fit model, these two values are presumed to be close, often with a $R^{2}$score of training set slightly higher than that of the testing set. Based on this strategy, we found that $n_{c}=4$ is the best value for the current study ($R^{2}\text{score}=0.68$ for test data set, $R^{2}\;\text{score}=0.72$ for training dataset). Leveraging more data and applying advanced prediction methods, we expect to see an increase in this number in our future work).

Despite the majority of published works that use PCA routines for SSA, we used PLS as a *supervised* order reduction tool to guarantee the maximum correlation between the extracted features and the output. The key difference between PCA and PLS is that the former is unsupervised, meaning that it is applied without the consideration of the correlation between the inputs and outcome, but aims to maximize the variance among the input data to make them as much separatable as possible. On the other hand, PLS reduces the order by considering both input and output (Figure 1) and is based on maximizing the correlation between the extracted components and the output. As a clarifying example, consider a cohort of 1,000 individuals for which a set of information is given, that includes several tens of features for each subject, including the average number of smokes and average minutes of exercise per day. Assume we wish to apply an order reduction to feed a prediction model which aims to predict lung cancer in the next 10 years for each individual. If these persons have higher variety in their other features (likely unrelated to cancer), applying PCA will not assign heavy weights to smoke and exercise, but to features which exhibit higher varieties in their values’ distribution. However, applying PLS will probably consider much higher weights for these two values, due to the high correlation that likely will be observed between them and the output, that is the lung cancer occurrence.

### 4.1. Limitations and future directions

Although our novel approach promises insightful information for potential utility in clinical practice, it bears a number of limitations. The received $R^{2}$ score is 0.68, indicating that nonnegligible variability in the outcome data cannot be explained merely by this model. This score can be improved through two different strategies. While it should be noted that there is a limitation for the anatomy driven, latent information within the shapes to single-handedly explain the complex biological phenomenon of remodeling, using more advanced shape processing, encoding, and prediction algorithms may refine and enhance the information and improve the prediction performance. For instance, the prediction score may be improved by applying more advanced order reduction methods such as L-PLS. L-PLS is an extension of PLS regression, which aims to improve prediction by focusing not only on the covariance between the input and output, but also capturing the additional background information on the interdependence of the predictor variables (26). The predictor model could also be upgraded if the rather simple predictor engine used herein (i.e., SVR) could be replaced by deep neural networks capable of discovering more complicated relationships between the inputs and outputs (14,27).

Another approach to improve the prediction results is to include additional factors that contribute to the LV shape. The most important feature that merits inclusion in this and any physiological shape-based prediction is sex. In particular, it has been shown that left ventricle average shape varies in males and females (28) and distinguishing the shape categorization based on sex would refine our analysis. Thus, an idea for future extension of this work is to improve the prediction results by either introducing a new binary input to the predictor model to determine the sex or developing two independent SSM pipelines for male and female patients. The latter is expected to outperform the former since it provides two different average shapes for studied male and female cases and therefore defines a more meaningful average shape for each group. We postponed such an attempt in this work due to the small population of studied patients and lack of sufficient data for each sex. In a more comprehensive study, should a larger cohort of AS patients be available, other key-factors including diastolic disfunction, race, and comorbidities can also be incorporated to further improve the model outcome. It is worthy to mention that an alternative method to tackle shape features caused by race, sex, and other non-anatomical factors is by applying methods which aim to identify and eliminate confounding factors (29).

At the end, it is worthy to note that in any machine-learning-based approach the results will gain more reliability if the number of available samples increases. However, it can be mathematically explained that this is not a major concern in our case. While the size of the samples in our study is relatively small, we have extracted only five components out of the shapes, which is much smaller than the total number of samples. Moreover, we have as well used a prediction method with a very few tunable parameters which demands smaller sample size to achieve a satisfactory outcome. This is in contrast to artificial neural networks with several hidden layers each containing multiple nodes that, having multi-tens of parameters to tune, may require a sample size of over thousands to be accurately trained.

## Conclusion

With daily development of advanced mathematical models and machine learning algorithms in addition to augmented computational power and data analysis capabilities, it is expected that novel modeling approaches will be leveraged to serve clinical research and improve therapy especially for recent surgical methods like TAVR. We developed an SSA approach to estimate LVMI regression one year after TAVR based on patient-specific clinical images at the baseline. The LV geometry automatically generated by our developed deep learning platform is all our trained model requires to offer the desired outcome with compelling accuracy. Our validated platform with its novel reduced order approach enables real-time one-year LVMI regression estimation and promises a great potential to update the risk stratification and surgery planning guidelines based on predicted clinical outcomes.

## Data Availability

The datasets presented in this article are not readily available because medical images and follow up data are restricted by the IRB. Requests to access the datasets should be directed to frikhtegarnezami@bwh.harvard.edu.
